# Lisch nodules and iris mammillations in two siblings with familial legius syndrome

**DOI:** 10.1002/ccr3.2861

**Published:** 2020-09-01

**Authors:** Kaitlyn D. Bixel, Miguel J. Cano, Damon M. Johnson, Benjamin Gomez, Laura V. Lobsinger, Frank E. Valentin, David T. Hsieh, Luis O. Rohena

**Affiliations:** ^1^ Department of Pediatrics Brooke Army Medical Center JBSA – Fort Sam Houston San Antonio Texas; ^2^ Department of Ophthalmology Brooke Army Medical Center JBSA – Fort Sam Houston San Antonio Texas; ^3^ Division of Child Neurology Department of Pediatrics Brooke Army Medical Center JBSA – Fort Sam Houston San Antonio Texas; ^4^ Division of Medical Genetics Department of Pediatrics Brooke Army Medical Center JBSA – Fort Sam Houston San Antonio Texas; ^5^ Division of Medical Genetics Department of Pediatrics University of Texas Health Science Center at San Antonio San Antonio Texas

**Keywords:** café‐au‐lait macules, legius syndrome, lisch nodules, neurofibromatosis type 1, neurofibromatosis type 1‐like syndrome, NF1, SPRED1

## Abstract

Legius syndrome is characterized by numerous café‐au‐lait macules and intertriginous freckling, but typically lacks the distinctive tumor manifestations of neurofibromatosis type 1. We report two siblings with Legius syndrome and Lisch nodules illustrating the importance of eye surveillance in these patients.

## Graphical abstract

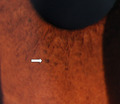

## 1 INTRODUCTION

Legius syndrome is a recently described genetic syndrome that is characterized by multiple café‐au‐lait macules (CALMs) and can also include intertriginous freckling, lipomas, macrocephaly, and neurobehavioral disorders. Legius syndrome is caused by a mutation in the *SPRED1* gene on chromosome 15 and is inherited in an autosomal dominant fashion. It is a rare disorder; the overall prevalence is unknown at this time, and most medical knowledge is based off of roughly 200 individuals with a genetically confirmed diagnosis.^1^, ^2^

Legius syndrome is also known as neurofibromatosis type 1‐like syndrome, as the initial clinical presentation of Legius syndrome can be clinically indistinguishable from neurofibromatosis type 1 (NF1). However, the lifetime clinical courses and prognoses for these diseases are very different. NF1 is characterized by development of multiple types of benign growths later in life, including neurofibromas and Lisch nodules; therefore, Legius syndrome is usually clinically differentiated from NF1 by the absence of these tumors.^1^, ^2^

We report on a familial case of three patients with molecularly confirmed Legius syndrome, in which two female siblings were discovered to have Lisch nodules on slit‐lamp examination. All three patients also demonstrated the characteristic phenotypic findings of numerous café‐au‐lait macules and skinfold freckling, and two presented with macrocephaly. None of the patients have presented any evidence of neurofibromas or other benign tumors except for the benign lisch nodules.

## 2 CASE REPORTS

### 2.1 Case 1

Our index patient was an 11‐year‐old female who presented to genetics clinic with concern for NF1. She was noted to have numerous café‐au‐lait macules and was additionally referred for ophthalmology evaluation. Her medical history consisted of a full‐term singleton birth complicated by gestational diabetes. Her vision and hearing were normal, and she was otherwise healthy without chronic medical problems or surgeries. She was developmentally appropriate and doing well in regular classes in the 5th grade. A previous MRI study of the brain and orbits was normal without evidence of optic pathway gliomas. Her family history revealed multiple CALMs in her father, older sister, and paternal aunt. Her father and older sister also had axillary freckling and macrocephaly. There was no family history of birth defects, genetically confirmed NF1, other genetic disorders, recurrent miscarriages, or consanguinity.

**Figure 1:**
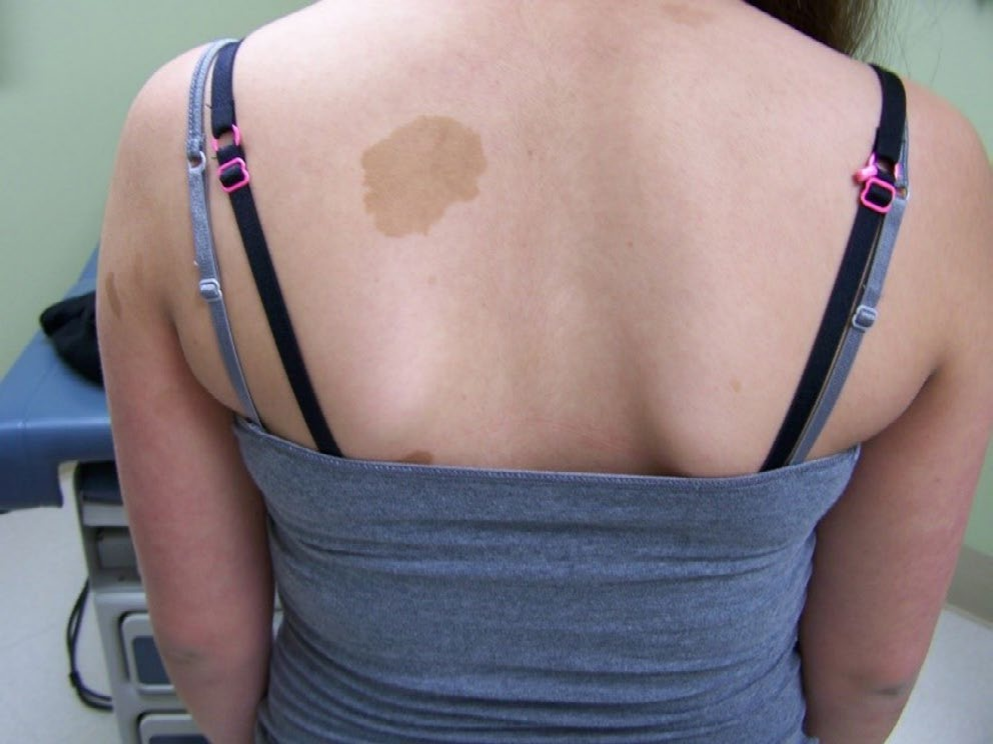
Multiple large café‐au‐lait macules in case 1

**Figure 2:**
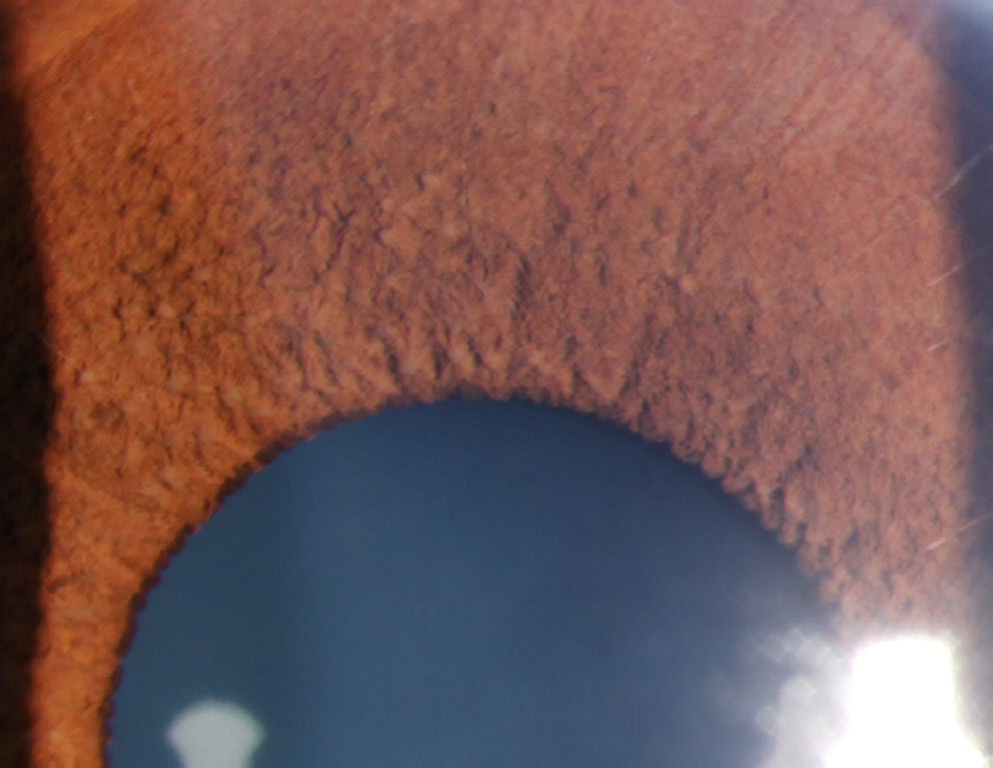
Lisch nodules and iris mammillations in right iris of case 1

**Figure 3:**
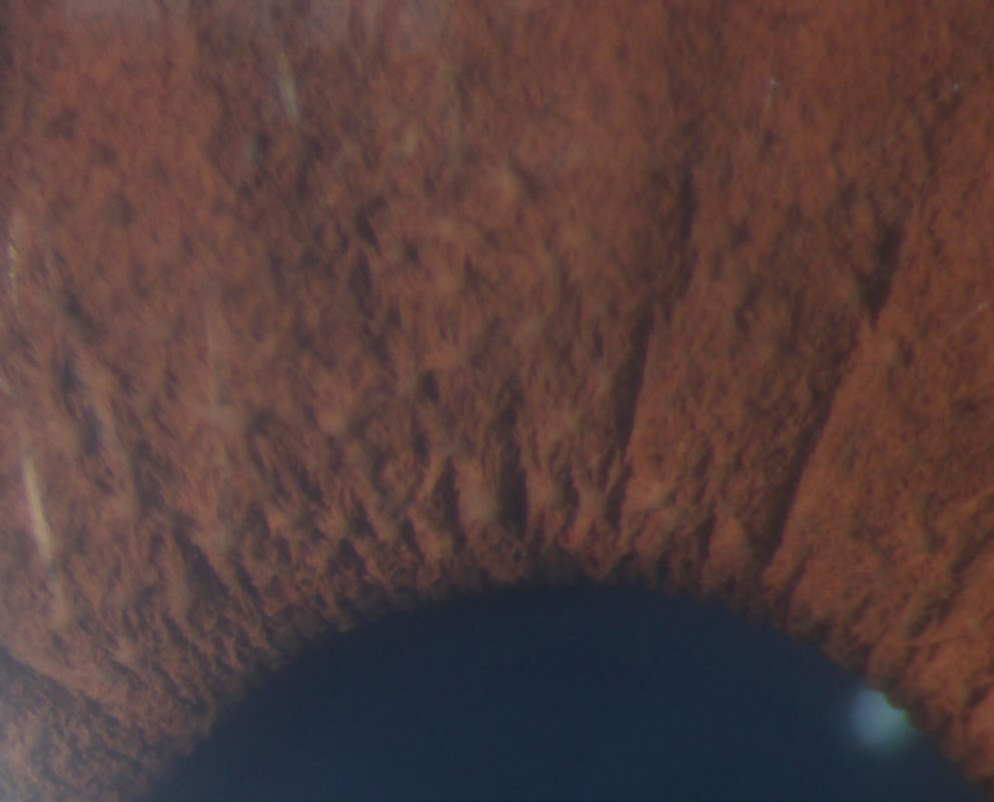
Left iris of case 1

Examination was notable for more than 5 CALMs larger than 1.5 cm (Figure 1), prominent axillary freckling, and an OFC at the 80th percentile. Slit‐lamp examination revealed multiple bilateral Lisch nodules and iris mammillations without optic pathway gliomas or cataracts (Figures 2 and 3). These were reviewed by multiple ophthalmologists and a neuro‐ophthalmologist and all are in agreement that Lisch nodules are present along with iris mammillations. No neurofibromas were noted.

### 2.2 Case 2

Our second patient was the 14‐year‐old sister of case 1. She was referred for genetic consultation after her sister was diagnosed with Legius syndrome. She was also noted to have numerous CALMs and intertriginous freckling, but an otherwise unremarkable medical history. A previous MRI study of brain and orbits was normal without evidence of optic pathway gliomas. She was developmentally appropriate and had high achievement in school through the 9th grade, including several advanced classes.

**Figure 4:**
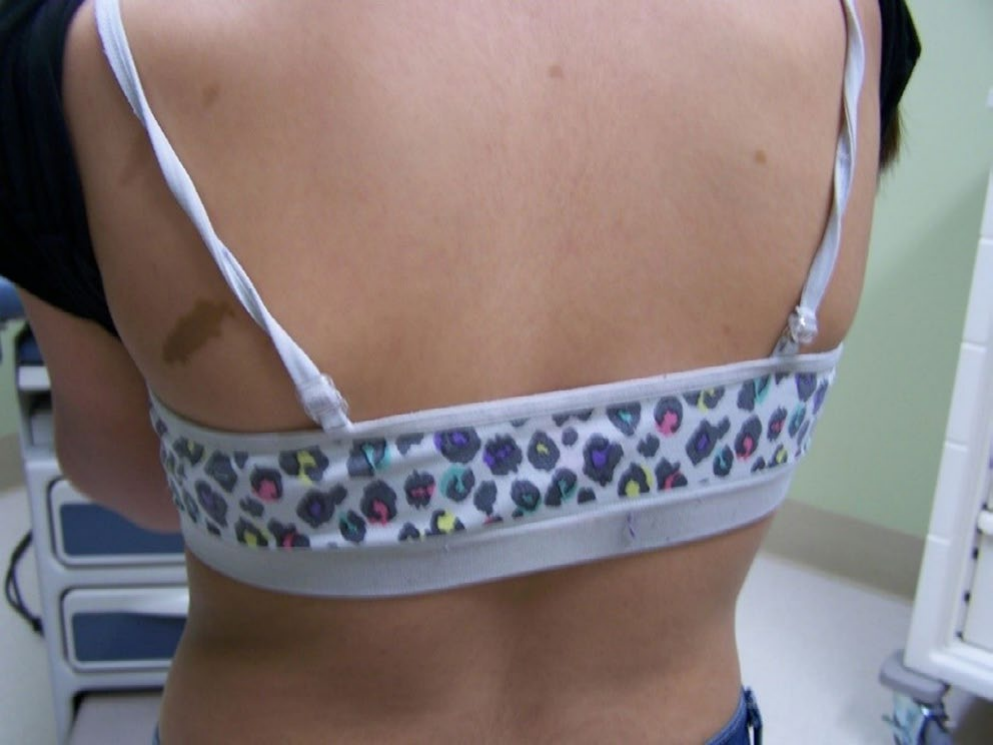
Multiple large café‐au‐lait macules in case 2

**Figure 5:**
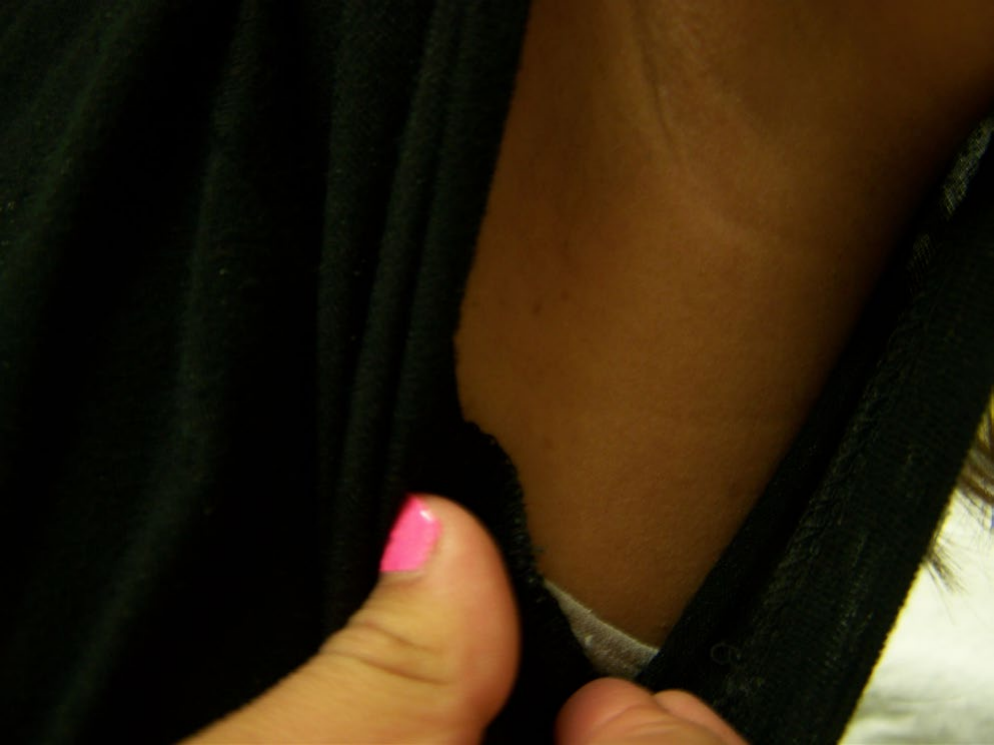
Intertriginous freckling in case 2

**Figure 6:**
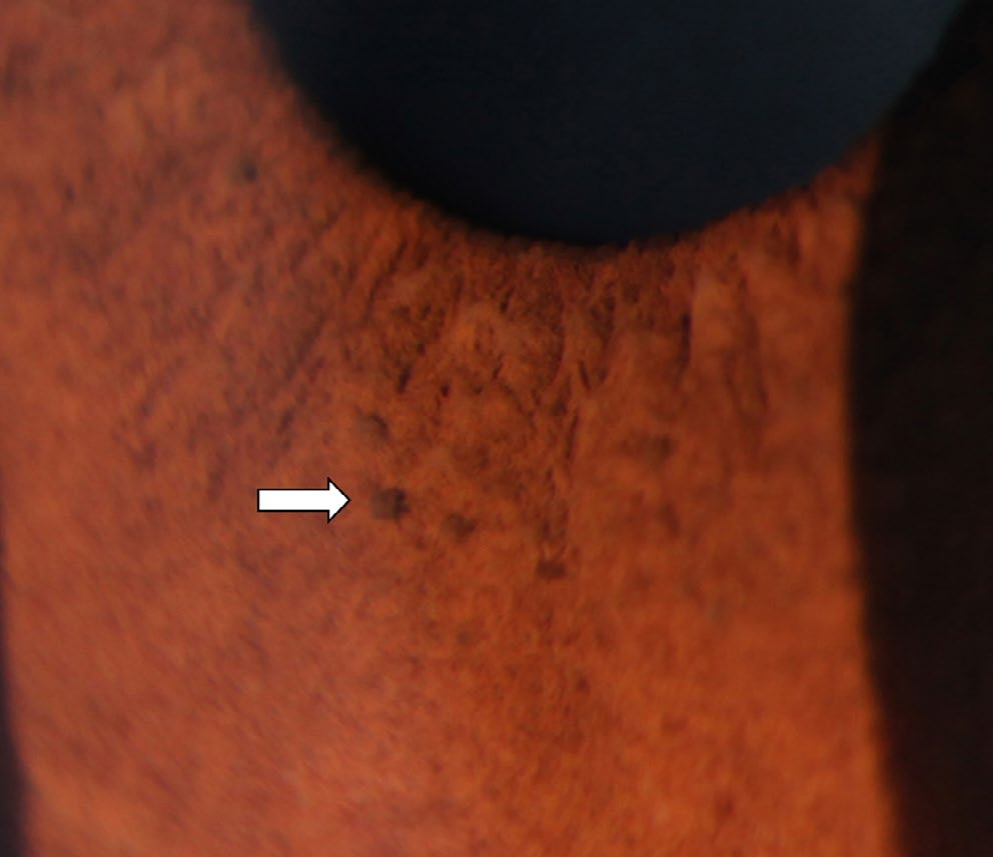
Right eye Lisch nodules in case 2

**Figure 7:**
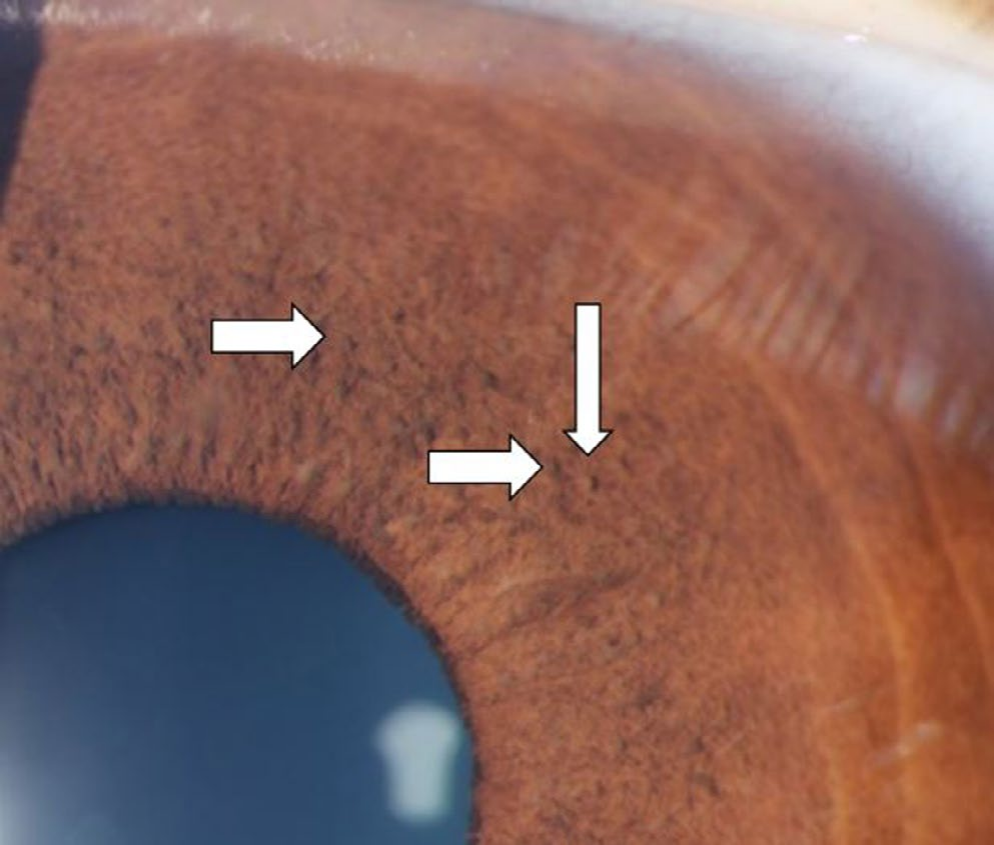
Left eye Lisch nodules in case 2

Examination revealed macrocephaly with OFC in the 98th percentile, large CALMs that were too numerous to count, and minimal axillary and intertriginous freckling (Figures 4 and 5). No neurofibromas were noted. She was referred to ophthalmology, where slit‐lamp examination also revealed bilateral Lisch nodules and iris mammillations, as it had in case 1 (Figures 6 and 7).

### 2.3 Case 3

The 49‐year‐old father of cases 1 and 2 also presented to genetics for evaluation after his daughters were diagnosed with Legius syndrome. Examination revealed macrocephaly with OFC of 60 cm, axillary freckling and more than 5 CALMs, present since childhood. He also underwent slit‐lamp examination by an experienced ophthalmologist, which did not reveal any Lisch nodules or iris mammillations.

### 2.4 Genetic Analysis

**Figure 8:**
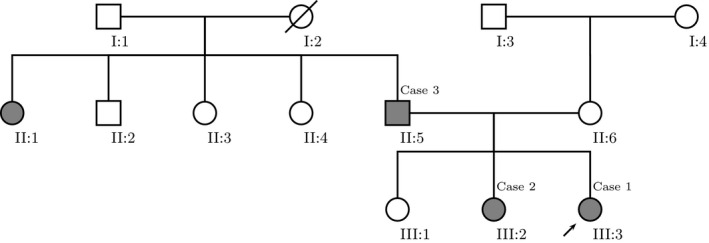
Pedigree of family. 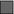 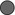 Affected male/ affected female (multiple CALMs). 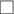 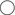 Unaffected male/ unaffected female. 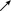 Proband. 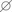 Deceased

A family pedigree is shown in Figure 8, indicating multiple family members affected by numerous CALMs*. NF1* gene sequencing, deletion, and duplication analysis were negative for the index case. A second test examining the *SPRED1* gene region identified a positive pathogenic variant in the *SPRED1* gene. It was caused by a sequence deletion of 2 nucleotides from exon 7 of the *SPRED1* mRNA (c.1151_1152delAG;p.Glu384Glyfs*5) causing a frameshift which created a premature translational stop signal. This specific mutation has not been previously reported in the literature.^3^, ^4^ *SPRED1* testing was sent for case 2 and case 3, and identified the same pathogenic variant found in case 1. The patient in case 3 also had a third daughter, who did not have phenotypic manifestations of Legius syndrome. She was also tested for *SPRED1* mutations, but none were identified.

## 3 DISCUSSION

There are several important points to be made regarding this case. First, for the clinical provider, all patients with multiple café‐au‐lait macules must be carefully evaluated. Although it can be an isolated cutaneous finding, six or more CALMs indicate significant risk for underlying genetic syndrome. A prospective cohort study of 41 children presenting with six or more CALMs found approximately 80% were eventually diagnosed with an associated genetic syndrome, with 58% of the cohort being diagnosed with NF1.^5^ Additionally, although NF1 is the most common diagnosis associated with multiple CALMs and should be considered in every patient presenting with these, the full differential is quite broad. It is therefore important to take a thorough history and examine the patient carefully for signs of any underlying genetic syndrome, with appropriate referrals to ophthalmology, genetics, and other subspecialties, as indicated.^5^, ^6^ Table 1 lists several syndromes that are strongly associated with café‐au‐lait macules, but it is by no means an exhaustive differential.

**Table 1:** Syndromes associated with multiple café‐au‐lait macules^5^, ^6^

Syndromes associated with multiple café‐au‐lait macules
Neurofibromatosis Type 1
Neurofibromatosis Type 2
Legius (NF1‐like) syndrome
Constitutional Mismatch Repair Deficiency syndrome (CMMR‐D)
McCune‐Albright syndrome
LEOPARD (multiple lentigenes) syndrome
Ring chromosome syndromes
Cowden (multiple hamartoma) syndrome
Noonan syndrome
Bannayan‐Riley‐Rulvalcaba syndrome
Familial multiple café‐au‐lait
Segmental neurofibromatosis

Second, this is the first report of Lisch nodules in patients with Legius syndrome.^1^, ^2^ Lisch nodules are melanocytic iris hamartomas and are a common ophthalmologic finding in NF1. Their prevalence increases with age and they occur in almost all patients with NF1 by the age of 21.^7^ Multiple Lisch nodules are pathognomonic of NF1, as it is rare for Lisch nodules to occur in the absence of other features of NF1^8^; however, several cases have been reported in the literature.^9^, ^10^

Lisch nodules must be carefully distinguished from other iritic findings, including iris mammillations, multiple iris nevi, iritic cysts, Brushfield spots, and malignancies.^8^, ^11^ Principally, the alternative diagnoses of iris mammillations and multiple iris nevi were considered in our cases. Iris mammillations are regularly spaced, deep brown iris elevations associated with oculodermal melanosis. They are described as pointed or conical lesions that form across the iris, likened to “goose‐skin” or a “field of stubble” by some early ophthalmologists.^11^ Iris nevi are typically flat, but can be slightly raised, and are darkly pigmented with indistinct margins. Lisch nodules are described as elevated and dome‐shaped with well‐defined borders, projecting from the iris surface. They can be clear to yellow or brown in color.^7^, ^9^, ^11^ Multiple experienced pediatric ophthalmologists were consulted and agreed that the lesions in cases 1 and 2 appear most consistent with a combination of iris mammillations and Lisch nodules. As Legius syndrome was first described little over a decade ago, and our body of knowledge continues to expand, these cases may demonstrate that the full phenotypic spectrum of Legius syndrome has not yet been defined.

Third, in following current NIH clinical diagnostic criteria for NF1 (Table 2), there exists the potential for misdiagnosis of patients with Legius syndrome.^12^ In fact, case 1 was initially misdiagnosed as NF1 by the clinical provider using these criteria. Compared with Legius syndrome, NF1 is relatively common, with an estimated population prevalence of 1 in 2500 to 3500.^8^, ^13^ The NIH criteria are currently being updated and are forthcoming.

**Table 2:** Criteria for NF1, data from the National Institutes of Health^12^

National Institutes of Health Diagnostic Criteria for NF1
At least two of the following clinical features must be present:
Six or more café‐au‐lait macules of more than 5 mm in greatest diameter in prepubertal individuals and more than 15 mm in greatest diameter in postpubertal individuals
Axillary freckling or freckling in the inguinal region
Optic glioma
Two or more neurofibromas of any type or one plexiform neurofibroma
Two or more iris hamartomas (Lisch nodules)
A distinctive osseous lesion (eg, sphenoid dysplasia or tibial pseudoarthrosis)
NF1 in a first‐degree relative, diagnosed by the above criteria

Patients who meet clinical diagnostic criteria for NF1 do not necessarily undergo molecular testing as part of their routine care, given the low likelihood of Legius syndrome or other alternative diagnosis, and the cost and complexity of genetic testing.^4^ NF1 is due to a germ‐line inactivating mutation in the *NF1* gene on chromosome 17 and genetic testing for NF1 is challenging because of the large size of the *NF1* gene, but current techniques have allowed mutations in this region to be identified approximately 95% of the time for patients with a clinical diagnosis of NF1.^8^, ^14^ Furthermore, prescribing a diagnosis of NF1 to a patient with Legius syndrome assigns a much poorer prognosis and does a disservice to the families affected by this syndrome. If a clear clinical diagnosis of NF1 is made, where features other than skin findings are used for diagnosis of NF1, molecular testing is not needed unless being obtained for family planning. Subsequently, if tests for *NF1* gene mutations are negative, we recommend that patients with a clinical diagnosis of NF1 undergo *SPRED1* testing or they can be done together as part of a next‐generation sequencing panel. Finally, we recommend a baseline ophthalmologic evaluation in all patients with a diagnosis of Legius syndrome.

## CONFLICT OF INTEREST

The authors have no conflict of interest to declare.

## AUTHOR CONTRIBUTIONS

KDB: primary author of the final manuscript. MJC: co‐authored manuscript. DMJ, BG, LVL, and FEV: contributed ophthalmological expertise and images. DTH: performed neurologic evaluation, MRI interpretation, and final manuscript editing. LOR: performed genetics evaluation, advised, and oversaw manuscript drafting and publishing.

## DISCLOSURE

The view(s) expressed herein are those of the author(s) and do not reflect the official policy or position of Brooke Army Medical Center, the U.S. Army Medical Department, the U.S. Army Office of the Surgeon General, the Department of the Army, the Department of the Air Force and Department of Defense or the U.S. Government.
